# In-silico based identification and functional analyses of miRNAs and their targets in Cowpea (*Vigna unguiculata* L.)

**DOI:** 10.3934/genet.2017.2.138

**Published:** 2017-06-29

**Authors:** Zareen Gul, Muhammad Younas Khan Barozai, Muhammad Din

**Affiliations:** Department of Botany, University of Balochistan, Sariab Road, Quetta, Pakistan

**Keywords:** microRNAs, conserved nature, cowpea (*Vigna unguiculata* L.), homology search

## Abstract

Cowpea (*Vigna unguiculata* L.) is an important leguminous plant and a good diet due to presence of carbohydrate and high protein contents. Currently, only few cowpea microRNAs (miRNAs) are reported. This study is intended to identify and functionally analyze new miRNAs and their targets in cowpea. An in-silico based homology search approach was applied and a total of 46 new miRNAs belonging to 45 families were identified and functionally annotated from the cowpea expressed sequence tags (ESTs). All these potential miRNAs are reported here for the first time in cowpea. The 46 new miRNAs were also observed with stable hairpin structures with minimum free energy, ranging from −10 to −132 kcal mol^−1^ with an average of −40 kcal mol^−1^. The length of new cowpea miRNAs are ranged from 18 to 26 nt with an average of 21 nt. The cowpea miRNA-vun-mir4414, is found as pre-miRNA cluster for the first time in cowpea. Furthermore, a set of 138 protein targets were also identified for these newly identified 46 cowpea miRNAs. These targets have significant role in various biological processes, like metabolism, transcription regulation as transcription factor, cell transport, signal transduction, growth & development and structural proteins. These findings are the significant basis to utilize and manage this important leguminous plant-cowpea for better nutritional properties and tolerance for biotic and abiotic stresses.

## 1. Introduction

MicroRNAs (miRNAs) are distinctive regulatory member of the small RNAs that regulate gene silencing at post-transcriptional level. Gene silencing by miRNAs is an important, advance and exciting area of present regulatory RNA research. They are endogenous, non-coding in nature and about 18 to 26 nucleotides (nt) in size. They are the negative regulator at post-transcriptional stage of gene regulation [1]. Initially, a self-folded stable hair-pin/stem-loop secondary structure termed as precursor-miRNAs (pre-miRNAs) is generate from long single strand RNA known as primary miRNA (pri-miRNA). Later the pre-miRNAs give rise a small sized (18–26nt) functional RNA known as mature miRNA. This mature miRNA is integrate into argonaute protein and advanced into the RNA induced silencing complex (RISC) [2],[3]. The RISC complex having mature miRNA triggers post-transcriptional gene suppression of the messenger RNA (mRNA) either by inhibiting protein encoding or by activating mRNA degradation. This inhibition and degradation capability of the miRNA depends on the scale of complementarity between miRNA and its targeted mRNA [4]. In case of partial pairing between miRNAs and its mRNA target causes its inhibition. While, the complete pairing of miRNAs with it mRNA target causes the mRNAs degradation [1],[5]. They participate as gene regulator in almost each and every life activity, such as growth and development, foreign genes suppression, signal transduction, environmental stresses and as a defense against the attacking microbes in various living organisms [1],[6]–[9]. Majority of the miRNAs show conserved behavior among various plant species. Many researchers, based on this conserved nature, have identified a huge number of miRNAs using comparative genomic approaches in a wide range of plant species, including cowpea [10], *Brassicanapus* [11], *Glycinemax* [12], cotton species [13],[14], *Zeamays* [15], tobacco [16], switch grass [17], Phaseolus [18], tomato [19], eggplant [20] and chilli [21]. These reports strongly suggest that comparative genomic strategies are valid, highly efficient, convenient, and economical-friendly methods to identify new miRNAs.

Cowpea (*Vigna unguiculata* L.) is an important leguminous crop of Asia, Africa, Southern Europe and USA [22]. It is a good food due to the presence of carbohydrate and high protein contents. This makes it not only essential diet to the human, but also serve as fodder to livestock. Cowpea is also significant to grow under low soil fertility, heat and drought. It is a key constituent of low-input farming systems for farmers. Cowpea also play vital role in the nitrogen fixation which is necessary for the enhancement of soil productiveness [22],[23]. Very little reports and data are available about the miRNAs in this important plant. According to the latest version of miRNA registry database (Version Rfam 21.0, released June, 2014) [24], only few miRNAs are available for cowpea. This situation demands to focus and profile new miRNAs and their targets in cowpea that will act as preliminary data to manage and understand the cowpea at molecular level.

Consequently, a total of 46 new miRNAs belonging to 45 families in cowpea were identified. In this study, one miRNA gene was also found as pre-miRNA cluster (vun-mir4414). Furthermore, these newly identified miRNAs were also validated for their protein targets.

## 2. Materials and methods

### 2.1. Identification of raw sequences

A similar methodology [15] with a little modification as described by Barozai MYK, et al. [13] was applied to profile the potential miRNAs from cowpea expressed sequence tags (ESTs). As reference miRNAs, a total of 4739 known plant miRNA sequences, both precursors and matures, were downloaded from the microRNA registry database (Version Rfam 21.0 released June, 2014) [24], and subjected to basic local alignment search tool (BLAST) for alignment against publicly available 187487 ESTs of cowpea from the dbEST (database of EST), release 130101 at http://blast.ncbi.nlm.nih.gov/Blast.cgi, using BLASTn program [25].

### 2.2. Creation of single tone EST

The repeated ESTs from the same gene were eliminated and a single tone EST per miRNA was produced by using BLASTn program against the cowpea EST database with default parameters [25].

### 2.3. Elimination of coding sequences

The initial potential miRNA sequences of cowpea, predicted by the mature source miRNAs, were checked for protein coding. The FASTA format of initial potential sequences were subjected against protein database at NCBI using BLASTX with default parameter [26] and the protein coding sequences were removed.

### 2.4. Creation of hair-pen structures

The initial potential candidate cowpea miRNA sequences, confirming as non-protein coding nature, having 0–4 mismatches with the reference miRNAs and representing single tone gene were subjected to generate hair-pen or secondary structures. Publicly available Zuker folding algorithm http://www.bioinfo.rpi.edu/applications/mfold/rna/form1.cgi, known as MFOLD (version 3.6) [27] was used to predict the secondary structures. The MFOLD parameters were adjusted same as published by various researchers for the identification of miRNAs in various plant and animal species [7],[8],[28]. For physical scrutinizing, the hair-pen structures either showing the lowest free energy ≤−18 kcal mol^−1^ or less than or equal to the lowest free energy of the reference miRNAs were preferred. The Ambros et al. [29] threshold values were applied as reference to finalize the potential miRNAs in cowpea. The stem regions of the stem-loop structures were checked and confirmed for the mature sequences with either at least 16 or equal to the reference miRNAs base pairing involved in Watson-Crick or G/U base pairing between the mature miRNA and the opposite strand (miRNA*).

### 2.5. Convergence and phylogenetic analysis

The convergence and phylogenetic analysis was carried out for the one of conserved cowpea miRNA (vun-mir398). Simply, the vun-mir398, for its conserved behavior in different plant species was checked for convergence and phylogenetic investigation. The vun-mir398 alignment was created with *Glycine max* (gma), *Nicotiana tabacum* (nta) and *Cucumis melo* (cme) by the publicly accessible web logo: a sequence logo generator and ClustalW to produce cladogram tree using neighbor joining clustering method respectively. The results were saved.

### 2.6. Prediction of miRNAs targets

Dual schemes were used to predict the potential targets for cowpea miRNAs. In the first scheme, the newly identified cowpea miRNAs were subjected to psRNATarget (http://bioinfo3.noble.org/psRNATarget), with default parameters [30]. The cowpea miRNAs that not produced potential targets through psRNATarget, were subjected to the second scheme as described by Barozai [31]. Briefly, the cowpea mature miRNA sequences were subjected as queries through BLASTn program. The parameters were adjusted as, database: reference mRNA sequences (refseq_rnat); organism: *Vigna unguiculata* (taxid:4072) and Program Selection: highly similar sequences (megablast). The mRNA sequences showing ≥75% query coverage were selected and further subjected to RNA hybrid—a miRNA target prediction tool [32]. Only targets, confirming stringent seed site located at either positions 2–7 and/or 8–13 from the 5′ end of the miRNAs along with the supplementary site and having minimum free energy (MFE) ≤−20 kcal mol^−1^ were selected. For more stringency, these targets were subjected to the NTNU microRNA target prediction tool available at http://tare.medisin.ntnu.no/mirna_target/search#results, to confirm the RNA hybrid results. These predicted targets were further analyzed through Gene Ontology (GO) on AmiGO website.

## 3. Results and discussion

### 3.1. The new cowpea miRNAs

In order to identify and characterize the potential miRNAs in cowpea, a comparative genomic approach was applied using bioinformatics tools. This is in agreement with the previous reports [8],[28],[31] that the homology based search by applying comparative genomics is a valid and logical approach to find interesting findings in plants at genomic level. The current study resulted a total of 46 new conserved miRNAs from the analyses of 187487 cowpea ESTs using bioinformatics tools (Table 1). The 46 potential cowpea miRNAs belong to 45 families (vun-miR: 398, 413, 435, 834, 1512, 1514, 1525, 1848, 2095, 2606, 2609, 2622, 2630, 2636, 2657, 2678, 2950, 3434, 4351, 4392, 4408, 4414 (cluster), 4992, 4996, 5012, 5043, 5215, 5216, 5219, 5227, 5241, 5246, 5255, 5261, 5280, 5290, 5298, 5376, 5561, 5758, 5770, 6252, 7696, 8182, 9748). The vun-miR4414 family is observed as cluster pre-miRNA. Available miRNAs literature revealed that all these 46 miRNAs are profiled for the first time in cowpea. In the light of the empirical formula for biogenesis and expression of the miRNAs suggested by Ambros et al. [29], these miRNAs are considered as a valid candidate after justifying the criteria B, C and D. According to Ambros et al. [29] only the criterion D is enough for homologous sequences to validate as potential miRNAs in other species. The present study is in agreement with the other research groups [21],[33]–[36] where similarity based search by applying comparative genomics has produced novel and interesting findings in plants genomics.

**Figure 1.**
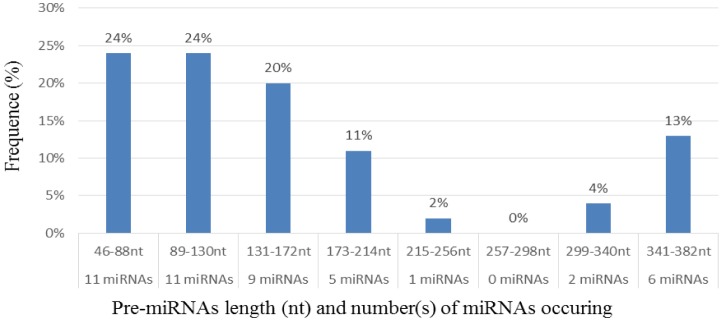
Distribution of the newly identified cowpea pre-miRNAs on the basis of their length.

**Table 1.** The newly identified conserved cowpea miRNAs characterization. Cowpea miRNAs were characterized in terms of precursor miRNA length (PL), minimum free energy (MFE), mature sequence (MS), number of mismatches (NM) (represented in bold red and enlarged font size), mature sequence length (ML), source EST (SE), mature sequence arm (MSA), GC content percentage (GC%), SL = Strand Location and organ of expression (OE).

vun miRNAs	Ref. miRNAs	PL	MFE	MS	NM	ML	SE #	MSA	GC%	SL	OE
vun-mir398	mtr-mir398a	131	−32.24	TGTGTTCTCAGGTC**G**CCCCT**G**	2	21	FF542932	5′	61.90	+	leaves
vun-mir413	ath-mir413	353	−88.55	TTAGTTTCTCTTGTTCTGC**TT**	2	21	FG940215	5′	33.33	+	mixed
vun-mir435	osa-mir435	347	−124.38	TTAT**GA**GG**CT**TTGGAGTTGA	4	20	FG811172	3′	40.00	+	mixed
vun-mir834	ath-mir834	135	−52.95	TGGTAGCAGT**G**GCGGTGGT**GG**	3	21	FG822669	3′	66.66	−	mixed
vun-mir1512	gma-mir1512a	46	−10.60	C**C**TTTAAGAATTTCA**-**TTA**--**	4	18	FG880488	3′	22.22	−	mixed
vun-mir1514	gma-mir1514	127	−31.70	TTCATTT**C**TAAAATAGGCAT**C**	2	21	FF388166	5′	28.57	−	root
vun-mir1525	gma-mir1525	78	−14.10	**G**GGGTTAA**A**TA**T**GTTTTTAGT	3	21	FG845219	5′	28.57	+	mixed
vun-mir1848	osa-mir1848	77	−32.20	C**G**CTCGCCGGCGCGCGCGT**C**CA	2	22	FG920123	3′	86.36	+	mixed
vun-mir2095	osa-mir2095	57	−17.20	CTTCCATTTATGA**C**A**T**GT**T**T	3	20	FG838629	5′	30.00	−	mixed
vun-mir2606	mtr-mir2606a	69	−13.00	T**T**GAAGT**GC**T**T**GGTTCTCACT	4	21	FG931806	5′	42.85	+	mixed
vun-mir2609	mtr-mir2609a	70	−13.00	T**T**GAAGT**GC**T**T**GGTTCTCACT	4	21	FG931806	5′	42.85	+	mixed
vun-mir2622	mtr-mir2622	210	−36.85	**C**TTGTGTGCCAT**T**GTGA**G**CTTA	3	22	FG900047	3′	42.85	−	mixed
vun-mir2630	mtr-mir2630a	114	−24.70	TGGTTTTGGTCT**T**TGGT**T**TT**A**	3	21	FF391380	5′	33.33	+	root
vun-mir2636	mtr-mit2636	191	−29.40	**GGAT**GTTAGTGTGCTGAATAT	4	21	FG814033	5′	38.09	−	mixed
vun-mir2657	mtr-mir2657	156	−35.38	T**T**TTATT**GT**AT**T**GATTTTGTTG	4	22	FG926034	5′	18.18	−	mixed
vun-mir2678	mtr-mir2678	136	−39.32	T**A**AA**G**TTGTTGCG**C**GTGTC	3	19	FF389500	3′	47.36	−	root
vun-mir2950	mes-mir2950	347	−83.20	TTCCATCTCTTGCA**G**ACTG**A**A	2	21	FG872933	5′	42.85	−	mixed
vun-mir3434	ath-mir3434	78	−17.40	T**G**AGAGTATCAGCCATG**A**GA	2	20	FF392538	3′	45.00	−	root
vun-mir4351	gma-mir4351	148	−63.30	**G**TT**A**GG**G**TTCAGTTGGAGTTGG	3	22	FG936300	3′	50.00	−	mixed
vun-mir4392	gma-mir4392	306	−80.53	TCTGTGAGAACGTGATTTCGGA	3	22	FG857306	5′	45.45	+	mixed
vun-mir4408	gma-mir4408	66	−20.70	**C**AACAACATTGGATGAG**TA**T**A**GGA	4	24	FG894682	3′	37.5	+	mixed
vun-mir4414a vun-mir4414b	mtr-mir4414a	120	−42.20	AGCTGCTGACTCGTTGGTTCA AT**T**CAACGATGCGGGAGCTGC	0 1	21 21	FF537171	5′ 3′	52.38 57.14	+ +	leaves
vun-mir4992	gma-mir4992	63	−21.20	**CA**TCTAAGATGGTTTTT**T**T**C**AG	4	22	FG926352	3′	31.81	−	mixed
vun-mir4996	gma-mir4996	163	−49.83	TAGAAG**T**T**A**CCCATGTTCTC	2	20	FF388735	3′	40.00	−	root
vun-mir5012	ath-mir5012	172	−43.44	TTTT**G**CTGCT**C**C**G**TGTGTTCC	3	21	FG809429	3′	52.38	+	mixed
vun-mir5043	gma-mir5043	125	−48.20	CTTCTCCTTCTCTGCACCACC	3	21	FG810406	5′	57.14	+	mixed
vun-mir5215	mtr-mir5215	181	−49.63	AGGAGGATGAGCTA**GT**TG**A**TT	3	21	FG939979	5′	42.85	+	mixed
vun-mir5216	mtr-mir5216a	124	−27.58	TT**G**GGAGTGAAAAAC**A**GTGGAA	2	22	FF399948	5′	40.90	+	root
vun-mir5219	mtr-mir5219	107	−25.23	TCATGGAATCTCAGCTGCAGCAG	1	23	FG850600	3′	52.17	−	mixed
vun-mir5227	mtr-mir5227	140	−18.04	**A**GAA**C**AGAAGAAGATTGA**A**GAA	3	22	FG915684	5′	31.81	−	mixed
vun-mir5241	mtr-mir5241a	381	−119.80	TG**GG**TGAATGGAAGAGTG**A**AT	3	21	FG904590	3′	42.85	+	mixed
vun-mir5246	mtr-mir5246	68	−18.70	**CAC**CAGA**G**AGCTTTGAAGGTT	4	21	FG856911	3′	47.61	+	mixed
vun-mir5255	mtr-mir5255	54	−10.40	TGAC**AG**GATAGAGGACATG**AC**	4	21	FG910302	5′	47.61	−	mixed
vun-mir5261	mtr-mir5261	311	−71.81	**CG**ATTGTAGATGGCTTTGGC**T**	3	21	FG838847	5′	47.61	−	mixed
vun-mir5280	mtr-mir5280	90	−20.22	TAA**G**TAGAAACGGGCCG**A**GAT**CG**GGG	4	26	FG915361	5′	57.69	−	mixed
vun-mir5290	mtr-mir5290	217	−30.24	AA**AG**T**A**GAGAGAGA**A**AGACACATA	4	24	FG852502	5′	33.33	+	mixed
vun-mir5298	mtr-mir5298a	192	−36.58	TGGAT**T**T**C**A**AG**ATGAAGATGAAGAA	4	25	FF402284	3′	32.00	−	root
vun-mir5376	gma-mir5376	341	−132.02	TG**G**AGATT**G**TGAAGAATTTG**A**GA	3	23	FG872123	3′	34.78	+	mixed
vun-mir5561	mtr-mir5561	346	−69.34	**A**TCT**C**TCTCTCTCTAAATG**T**A	3	21	FF390124	5′	33.33	−	root
vun-mir5758	mtr-mir5758	91	−22.60	TAAGTTGGA**TCT**ATGTATTTG	3	21	FG893334	3′	28.57	+	mixed
vun-mir5770	gma-mir5770a	98	−30.40	TTAGGACTATGGTTTGGA**T**GA	1	21	FG937135	3′	38.09	−	mixed
vun-mir6252	osa-mir6252	90	−20.90	AT**GA**GTTGT**G**TT**G**AGAGAGGGTT	4	23	FG841373	3′	43.47	−	mixed
vun-mir7696	mtr-mir7696a	173	−33.67	**A**CAAGT**A**CT**T**A**-**AATTCAAAA	4	20	FG864277	3′	20.00	−	mixed
vun-mir8182	ath-mir8182	170	−31.80	TTGTGTTGCGTTT**G**TG**A**TGA**C**T	3	22	FG942892	5′	40.90	−	mixed
vun-mir9748	gma-mir9748	98	−32.45	GAAGGAAGTGT**T**GAGGGA**G**GA**G**	3	22	FG921211	5′	54.54	+	mixed

**Figure 2.**
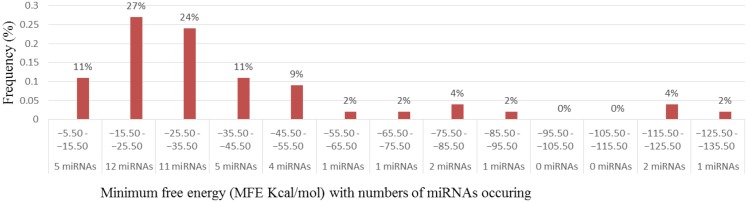
Distribution and classification of newly identified cowpea miRNAs on the basis of their minimum free energies (MFEs).

**Figure 3.**
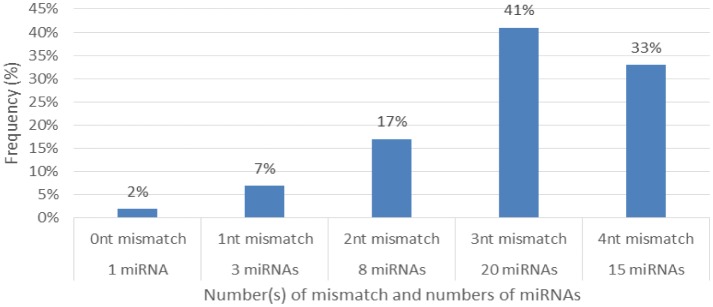
Distribution of the cowpea miRNAs mismatches (nt) with their reference miRNAs.

**Figure 4.**
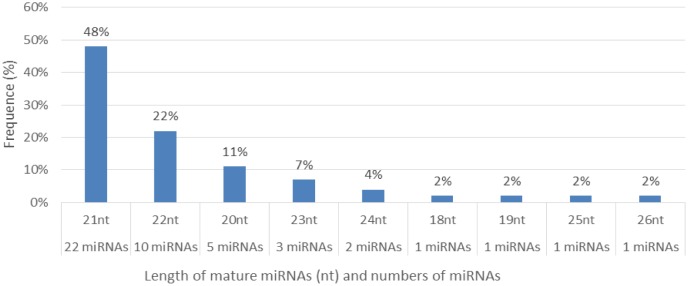
Distribution of the cowpea mature miRNAs for their length.

### 3.2. Characterization of cowpea miRNAs

**Figure 5.**
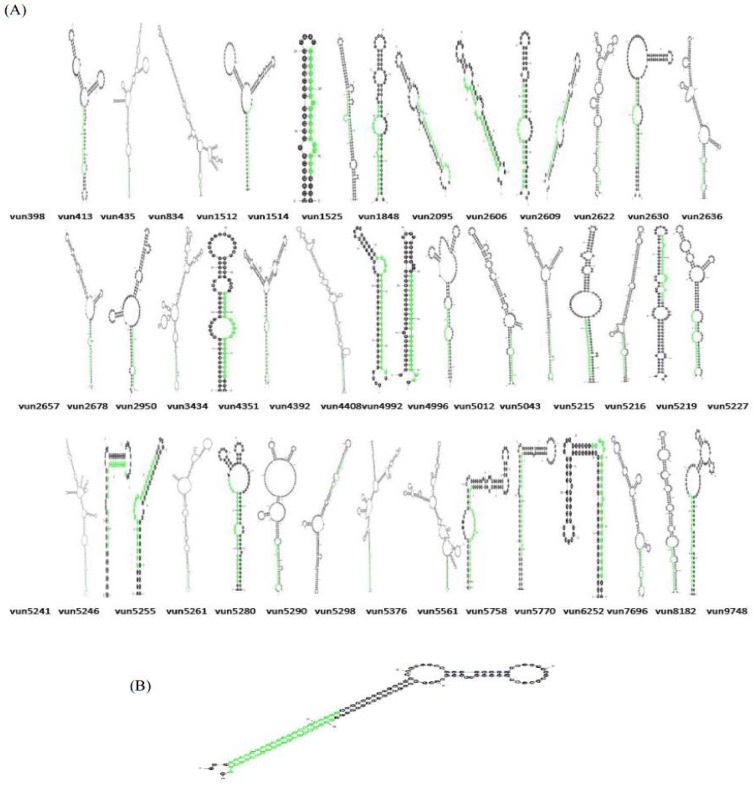
(A) The newly identified cowpea miRNAs' secondary structures. Cowpea pre-miRNAs secondary structures were developed through Mfold algorithm. These structures clearly showing the mature miRNAs in stem portion of the stem-loop structures. (B) Cowpea pre-miRNA cluster. Cowpea miRNA (vun-miR4414) was found as a pre-miRNA cluster with two mature miRNAs (miR4414a and miR4414b). The pre-miRNA cluster secondary structure was created by Mfold (version 3.6), showing mature sequences in green within the same pre-miRNA sequence

**Figure 6.**
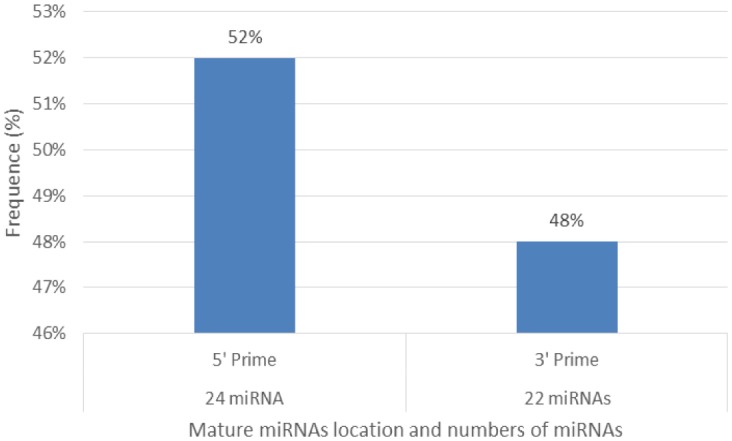
Distribution of mature miRNAs location on the either arms of hair-pen structures and numbers (frequency%) of miRNAs occurring.

**Figure 7.**
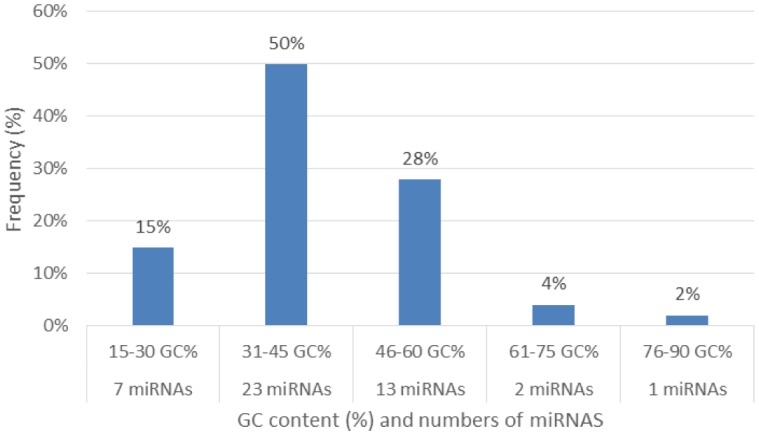
Percentage distribution of GC content and numbers (frequency%) of miRNAs occurring.

**Figure 8.**
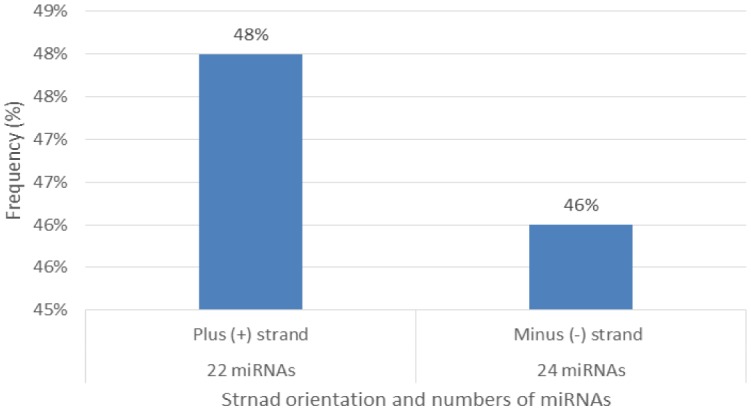
Percentage distribution of strand orientation and numbers (frequency%) of miRNAs occurring.

**Figure 9.**
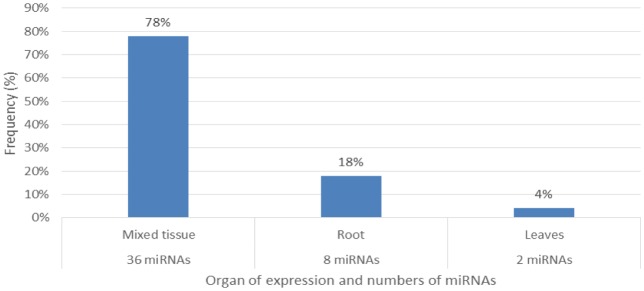
Percentage distribution of organ expression and numbers (frequency%) of miRNAs occurring.

**Figure 10.**
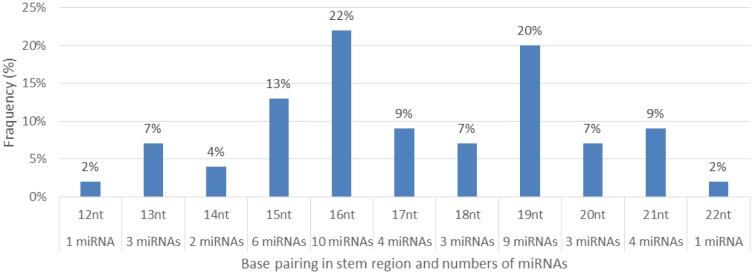
Percentage distribution of base pairing between the mature miRNA and the opposite arms (miRNAs*) in the stem region and numbers (frequency%) of miRNAs occurring.

Characterization of newly identified candidate miRNAs is a set crucial step for their validation, as reported earlier [16],[17],[37]. The pre-miRNA length of the profiled cowpea miRNAs ranges from 46 to 381 nt with an average of 159 nt. The pre-miRNAs were further illustrated on the basis of their length (Figure 1). The minimum folding free energy (MFE) of pre-miRNA is a vital and valid term of characterization. The newly identified potential cowpea pre-miRNAs have shown MFEs in range from −10 to −132 kcal mol^−1^ with an average of −40 kcal mol^−1^ as shown in Figure 2. The numbers of mismatches of mature sequences with their reference sequences were observed in a range of 0–4 with an average of three mismatches as categorized in Figure 3. These values are matched with the previously reported values in different plants [21],[37]–[39]. Mature miRNA sequences lengths were observed from 18 to 26 nt with an average of 21 nt as explained in Figure 4. These findings of mature sequences length are in agreement to prior published data in other plant species [16]–[18],[36]. The 52% cowpea miRNAs sequences were found at 5′ arm, while 48% were at 3′ arm (Figure 5(A),6). The GC content was found from 18 to 86% with an average of 42% as shown in Figure 7. Strand orientation is another important character for the generation of mature miRNAs transcripts. In this study, 24 mature miRNAs were found on minus strand while 22 were observed on plus strand of the transcripts (Figure 8). The same results for plus and minus strand orientation of mature miRNAs are in agreement with the earlier research work [40]. The identified conserved cowpea miRNAs were also characterized on the basis of their organ of expression as presented in Figure 9. These findings are similar with the earlier reports [37] and suggesting organ dependent expression pattern of miRNAs in cowpea. The miRNA organ specific expression would be utilized to manage the organogenesis in cowpea. The secondary self-folded stem-loop structures of the cowpea pre-miRNAs are observed with at least 17 nucleotides engaged in Watson-Crick or G/U base pairing between the mature miRNA and the opposite arms (miRNAs*) in the stem region (Figure 10). Except few where the reference miRNAs have also less base pairing and these precursors do not contain large internal loops or bulges. The mature miRNA sequences are observed in the double stranded stem region of the pre-miRNA secondary structures, as shown in Figure 5(A). Almost similar findings for various plant and animal species were reported by many researchers [16],[17],[20],[37],[41],[42]. Furthermore, the newly identified cowpea miRNAs were also confirmed as non-protein coding nature by showing no significant similarity with known proteins. This validation strengthens the expressed nature for computationally identified miRNAs as non-coding RNAs. Similar results were observed in various research papers by many groups [16],[43],[44].

### 3.3. Cluster pre-miRNA gene in cowpea

In animals, a large number of miRNAs have been found in clusters and have been predicted to have similar expression profiles and functions [45]. The miRNA clusters have rarely been detected in plants. They were first reported by Jones-Rhoades and Bartel [46]. In this study, we also identified one pre-miRNA (mir4414) as cluster in cowpea having two mature miRNAs within Figure 5(B). On the basis of current available literature, this miRNA family (miR4414) was found for the first time in cowpea as a cluster.

### 3.4. Convergence and phylogenetic studies

**Figure 11.**
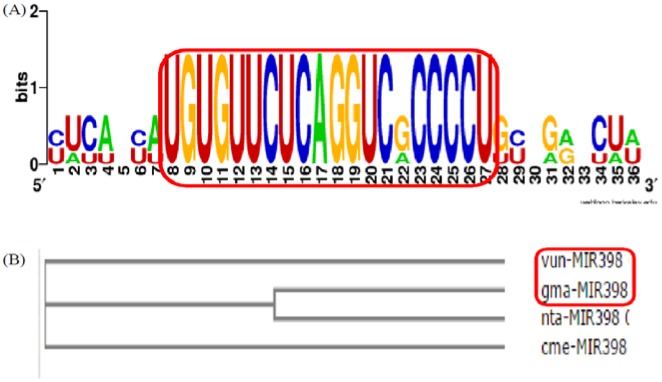
(A) Cowpea miRNA's conservation studies. Alignment of *V. unguiculata* (vun) miRNA (vun-mir398) with *G. max* (gma), *N. tabacum* (nta) and *C. melo* (cme) was generated using Web logo: a sequence logo generator, showing conserved nature mature miRNA sequences. The mature sequences highlighted in a rectangle red box. (B) Cowpea miRNA's phylogenetic analysis. *V. unguiculata* (vun) miRNA (vun-mir398) with *G. max* (gma), *N. tabacum* (nta) and *C. melo* (cme) was done with the help of ClustalW and cladogram tree was generated using neighbor joining clustering method. The phylogenetic tree showed that the *V. unguiculata* (vun) is more closed to *G. max* (gma) than *N. tabacum* (nta) and *C. melo* (cme). The closed plant species highlighted in a rectangle red box.

The newly characterized cowpea miRNA vun-mir398, due to its conserved nature, was investigated for convergence and phylogeny. Simply, the cowpea miRNA vun-mir398 alignment and cladogram tree, using neighbour joining clustering method, were created with *Glycine max* (gma), *Nicotiana tabacum* (nta) and *Cucumis melo* (cme) by the publicly available Web-Logo, a sequence logo generator [47] and ClustalW, a multiple sequence alignment tool [48]. The cowpea miRNA vun-mir398 is observed in convergence with *Glycine max* (gma), *Nicotiana tabacum* (nta) and *Cucumis melo* (cme) as shown in Figure 11(A). The Phylogenetic cladogram tree, as illustrated in Figure 11(B), clearly showed that on the basis of sharing a more recent common ancestor the cowpea miRNA is more closely related to *Glycine max* (gma) than *Nicotiana tabacum* (nta) and *Cucumis melo* (cme). Zeng et al. [49] have also reported conserved nature in Euphorbiaceous plants.

### 3.5. The potential cowpea miRNAs targeted genes

Profiling the potential cowpea miRNAs targeted genes is a vital step for validation of the computationally identified miRNAs. A total of 138 targeted genes were predicted for the 46 potential cowpea miRNAs. The detail description is mentioned in Table 2. Different cowpea miRNAs targeting same proteins and vice versa were predicted here. This showed that one miRNA target more than one mRNAs and a single mRNA targets by many miRNAs [50]. The profiled targeted genes are categories as, 27% (37 of 138) are engaged in metabolism, 26% (36 of 138) are playing role as transcription factors, 11% (15 of 138) are involved in transport activities, 11% (15 of 138) are shown with stress related, and the rest are engaged in hypothetical protein, signal transduction, growth and development, structural proteins and diseases related. Almost all of these targets were already reported as miRNA targets in other plants [7],[16],[17].

Majority (27%) of the newly characterized cowpea miRNAs are observed to regulate the metabolic proteins. Such findings regarding metabolism related genes targeted by miRNAs are similar with the prior publications in plants and animals [28],[43],[44]. Pectin methylesterase (PME) is an important enzyme that acts on pectin, a major component of plant cell wall. PME catalyzes reactions according to the double-displacement mechanism [51]. In this study, the PME is predicted as a putative target for vun-miR1882. Thus the vun-miR1882 is a valuable resource to regulate cell wall. Another important enzyme ribulose-1,5-bisphosphate carboxylase (Rubisco) is a key enzyme in photosynthesis and photorespiration, where it catalyzes the fixation of CO_2_ and O_2_, respectively. Due to its rate-limiting property in photosynthesis, it is the prime focus of improving the plant productivity [52]. The cowpea miRNA (vun-miR2657) is predicted to target this important enzyme which is the potential resource to modify Rubisco expression and ultimately plant productivity.

**Table 2.** Targets of cowpea miRNAs: As predicted by psRNAtarget and RNA hybrid in terms of miRNA family number, target acc., target description and function.

miRNA	Target Acc.	Target Description	Function	Alignment
vun-mir398	TC8412	Predicted protein	Hypothetical protein	miRNA 21 GUCCCCGCUGGACUCUUGUGU 1 ::::::::.:::: :::::: Target 24 CAGGGACGAUCUGAUAACACA 44
vun-mir413	TC18010	H/ACA ribonucleoprotein complex	Transcription factor	miRNA 21 UUCGUCUUGUUCUCUUUGAUU 1 ::::::::::::::::::::: Target 432 AAGCAGAACAAGAGAAACUAA 452
vun-mir413	FF538223	Tropinone reductase	Metabolism	miRNA 21 UUCGUCUUGUUCUCUUUGAUU 1 .:::::::.:: .:.:::::: Target 321 GAGCAGAAUAAUGGGAACUAA 341
vun-mir413	TC16544	Valyl-tRNA synthetase	Metabolism	miRNA 21 UUCGUCUUGUUCUCUUUGAUU 1 :.::::::::::.:::. ::: Target 1013 AGGCAGAACAAGGGAAGAUAA 1033
vun-mir413	TC9044	Uroporphyrinogen decarboxylase	Metabolism	miRNA 21 UUCGUCUUGUUCUCUUUGAUU 1 .:: :::: :::::::.::.: Target 59 GAGAAGAAGAAGAGAAGCUGA 79
vun-mir435	TC9534	Chromosome chr12 scaffold_238,	Hypothetical protein	miRNA 20 AGUUGAGGUUUCGGAGUAUU 1 :::::::::: :..::::.: Target 242 UCAACUCCAAUGUUUCAUGA 261
vun-mir435	FF387447	Chromosome chr9 scaffold_7,	Hypothetical protein	miRNA 20 AGUUGAGGUUUCGGAGUAUU 1 ::::.:.:::.::::: ::: Target 386 UCAAUUUCAAGGCCUCCUAA 405
vun-mir435	TC16349	Ripening related protein	Growth and development	miRNA 20 AGUUGAGGUUUCGGAGUAUU 1 :.::::::::: :.:.::.: Target 523 UUAACUCCAAAACUUUAUGA 542
vun-mir435	FG810938	Protein kinase	Signal transduction	miRNA 20 AGUUGAGGUUUCGGAGUAUU 1 ::: :::::: :::::.::. Target 474 UCACCUCCAAUGCCUCGUAG 493
vun-mir834	TC4272	SCOF-1	Transcription factor	miRNA 21 GGUGGUGGCGGUGACGAUGGU 1 :::::.::::::: :.::::: Target 281 CCACCGCCGCCACCGUUACCA 301
vun-mir834	TC8566	Cytochrome P450 monooxygenase CYP83E9	Metabolism	miRNA 20 GUGGUGGCGGUGACGAUGGU 1 ::::: : :::::::::::: Target 465 CACCAACACCACUGCUACCA 484
vun-mir834	TC7191	DnaJ-like protein	Stress related	miRNA 21 GGUGGUGGCGGUGACGAUGGU 1 ::.::::::::::::: :::. Target 173 CCGCCACCGCCACUGCAACCG 193
vun-mir834	FG876294	Zinc finger-like protein	Transcription factor	miRNA 21 GGUGGUGGCGGUGACGAUGGU 1 ::::::::::::: :: :::: Target 138 CCACCACCGCCACCGCCACCA 158
vun-mir834	TC4023	GroEL-like chaperone, ATPase	Stress related	miRNA 21 GGUGGUGGCGGUGACGAUGGU 1 :: ::.:::::.:::::.::: Target 78 CCUCCGCCGCCGCUGCUGCCA 98
vun-mir834	TC7031	Oxophytodienoate reductase	Metabolism	miRNA 21 GGUGGUGGCGGUGACGAUGGU 1 .::.::.:::::::::: ::: Target 19 UCAUCAUCGCCACUGCUUCCA 39
vun-mir834	TC15421	MYB	Transcription factor	miRNA 20 GUGGUGGCGGUGACGAUGGU 1 ..:.::.::.:::::::::: Target 955 UGCUACUGCUACUGCUACCA 974
vun-mir834	GH622195	Ribosomal protein	Structural protein	miRNA 21 GGUGGUGGCGGUGACGAUGGU 1 :::::.:::::::: ::::: Target 110 CCACCGCCGCCACUUCUACCU 130
vun-mir834	TC7768	Calcium-binding EF-hand)	Transcription factor	miRNA 21 GGUGGUGGCGGUGACGAUGGU 1 ..::.::.:..::::.::::: Target 470 UUACUACUGUUACUGUUACCA 490
vun-mir1512	XM_013230906	Biomphalaria glabrata dual oxidase	Metabolism	target 5′ C U 3′ AAUGAAAUUCUUAAAGG UUACUUUAAGAAUUUCC miRNA 3′ A 5′
vun-mir1512	XM_006957329	Nucleoside triphosphate hydrolase protein	Transcription factor	target 5′ U A 3′ UAAUGAAAUUCUUAAAG AUUACUUUAAGAAUUUC miRNA 3′ C 5′
vun-mir1512	KC463855	NB-LRR receptor (RSG3-301)	Transcription factor	target 5′ C CCC GG U 3′ AAUGA AA CUUGAAGG UUACU UU GAAUUUCC miRNA 3′ A AA 5′
vun-mir1512	EF076031	Phosphatidic acid phosphatase alpha (PAPa)	Metabolism	target 5′ A AAGGGG G A 3′ UGGUGAAA UC UAAAGG AUUACUUU AG AUUUCC miRNA 3′ A A 5′
vun-mir1512	AF413209	Dolichos biflorus chloroplast ribulose-1,5-bisphosphate carboxylase	Metabolism	target 5′ C G 3′ UGGUGAAAU UAAAGG AUUACUUUA AUUUCC miRNA 3′ AGA 5′
vun-mir1514	FF388166	NAC domain-containing protein 78	Transcription factor	miRNA 21 CUACGGAUAAAAUCUUUACUU 1 ::::::::::::::::::::: Target 687 GAUGCCUAUUUUAGAAAUGAA 707
vun-mir1514	FF540114	Phosphate transporter family protein	transporter	miRNA 20 UACGGAUAAAAUCUUUACUU 1 ::::::.::::.:::::::: Target 461 AUGCCUGUUUUGGAAAUGAA 480
vun-mir1514	TC15423	NAM-like protein	Transcription factor	miRNA 20 UACGGAUAAAAUCUUUACUU 1 ::::::.::::.:::::::: Target 589 AUGCCUGUUUUGGAAAUGAA 608
vun-mir1514	TC869	ATP-binding cassette sub-family f member 2	Transporter	miRNA 21 CUACGGAUAAAAUCUUUACUU 1 :: ::.:::: :::::::::: Target 733 GAGGCUUAUUCUAGAAAUGAA 753
vun-mir1514	FG830151	Starch branching enzyme	Metabolism	miRNA 20 UACGGAUAAAAUCUUUACUU 1 ::::: ::::::::.:::: Target 314 AUGCCAAUUUUAGAGAUGAU 333
vun-mir1514	TC5197	Cytochrome c biogenesis protein-like	Transporter	miRNA 20 UACGGAUAAAAUCUUUACUU 1 :: .::::::::::.:::: Target 749 AUAUCUAUUUUAGAGAUGAU 768
vun-mir1525	TC17248	Salt-tolerance protein	Stress related	miRNA 21 UGAUUUUUGUAUAAAUUGGGG 1 ::::::::::::::::::.:. Target 306 ACUAAAAACAUAUUUAACUCU 326
vun-mir1525	FG915097	UDP-N-acetylmuramoylalanine-D-glutamate ligase	Transcription factor	miRNA 21 UGAUUUUUGUAUAAAUUGGGG 1 ::::::::.::::::.:::: Target 468 ACUAAAAAUAUAUUUGACCCA 488
vun-mir1525	TC14268	Non-specific lipid-transfer protein	transporter	miRNA 20 GAUUUUUGUAUAAAUUGGGG 1 ::.:::...:::::::::.: Target 505 CUGAAAGUGUAUUUAACCUC 524
vun-mir1525	TC18336	Heat shock protein	Stress related	miRNA 20 GAUUUUUGUAUAAAUUGGGG 1 .:.:..:.::::::.::::: Target 166 UUGAGGAUAUAUUUGACCCC 185
vun-mir1848	EG424245	Radical SAM domain protein	Metabolism	miRNA 20 CUGCGCGCGCGGCCGCUCGC 1 :: ::: :::: :::::::: Target 110 GAAGCGAGCGCAGGCGAGCG 129
vun-mir2095	FF402667	Resistance protein MG55	Stress related	miRNA 20 UUUGUACAGUAUUUACCUUC 1 .: :.:::::::::::::: Target 592 GAUCGUGUCAUAAAUGGAAU 611
vun-mir2095	TC2784	Vacuolar protein sorting-associated protein 26-like protein	transporter	miRNA 20 UUUGUACAGUAUUUACCUUC 1 ::::::::::: .: ::::: Target 824 AAACAUGUCAUCGAAGGAAG 843
vun-mir2606	TC406838	SNF1 related protein kinase	Signal transduction	miRNA 20 CACUCUUGGUUCGUGAAGUU 1 : :::::. ::::::::::: Target 1051 GAGAGAAUAAAGCACUUCAA 1070
vun-mir2606	TC401737	ATP binding protein	Transcription factor	miRNA 20 CACUCUUGGUUCGUGAAGUU 1 :::::::.::::.::::: Target 242 UCGAGAACCGAGCAUUUCAA 261
vun-mir2606	NP305366	Hypothetical protein	Hypothetical protein	miRNA 21 UCACUCUUGGUUCGUGAAGUU 1 : ::::::.:.::.::::::. Target 420 ACUGAGAAUCGAGUACUUCAG 440
vun-mir2609	NP038997	Jasmonate induced protein	Stress related	miRNA 21 UCACUCUUGGUUCGUGAAGUU 1 : ::.:: ::::::::::::: Target 220 ACUGGGAUCCAAGCACUUCAA 240
vun-mir2609	NP568563	SEC14-like protein	Transcription factor	miRNA 21 UCACUCUUGGUUCGUGAAGUU 1 :: :::::::::: ::::.:: Target 417 AGCGAGAACCAAGGACUUUAA 437
vun-mir2609	TC406838	SNF1 related protein kinase-like protein	Signal transduction	miRNA 20 CACUCUUGGUUCGUGAAGUU 1 : :::::. ::::::::::: Target 1051 GAGAGAAUAAAGCACUUCAA 1070
vun-mir2609	TC401737	ATP binding protein	Signal transduction	miRNA 20 CACUCUUGGUUCGUGAAGUU 1 :::::::.::::.::::: Target 242 UCGAGAACCGAGCAUUUCAA 261
vun-mir2622	TC9003	Alpha-expansin 2	Metabolism	miRNA 22 AUUCGAGUGUUACCGUGUGUUC 1 :::::::::::::::::::::: Target 64 UAAGCUCACAAUGGCACACAAG 85
vun-mir2630	TC15462	Auxin influx transport protein	Transporter	miRNA 20 UUUUGGUUUCUGGUUUUGGU 1 ::::::::: ::::::::: Target 293 AAAACCAAAAACCAAAACCU 312
vun-mir2630	FF390661	Serine/arginine repetitive matrix 1	Transcription factor	miRNA 20 UUUUGGUUUCUGGUUUUGGU 1 ::::: ::: :::::::::: Target 349 AAAACAAAAAACCAAAACCA 368
vun-mir2630	FG865319	Monosaccharid transport protein	Transporter	miRNA 20 UUUUGGUUUCUGGUUUUGGU 1 :::.:::::::.::.:::: Target 109 UAAAUCAAAGACUAAGACCA 128
vun-mir2630	TC4441	Ras-related protein RAB8-1	Transcription factor	miRNA 20 UUUUGGUUUCUGGUUUUGGU 1 ::::.:::: :::::::::: Target 75 AAAAUCAAA-ACCAAAACCA 93
vun-mir2630	TC1550	Homeodomain leucine zipper protein HDZ3	Transcription factor	miRNA 21 AUUUUGGUUUCUGGUUUUGGU 1 :.::::..:. :::::::::: Target 1253 UGAAACUGAGAACCAAAACCA 1273
vun-mir2630	FC457466	Pseudouridylate synthase	Metabolism	miRNA 21 AUUUUGGUUUCUGGUUUUGGU 1 :::::. :..:::.::::::: Target 504 UAAAAUGAGGGACUAAAACCA 524
vun-mir2630	TC6720	Ubiquitin carrier protein	Transporter	miRNA 20 UUUUGGUUUCUGGUUUUGGU 1 :::::::::: ::::: .:: Target 685 AAAACCAAAGCCCAAAUUCA 704
vun-mir2636	TC7750	NADH-ubiquinone oxidoreductase chain 2	Metabolism	miRNA 21 UAUAAGUCGUGUGAUUGUAGG 1 :::::.::::::::::.: .: Target 225 AUAUUUAGCACACUAAUAAUC 245
vun-mir2636	FF537611	Na^+^/H^+^ antiporter	Metabolism	miRNA 20 AUAAGUCGUGUGAUUGUAGG 1 : :::::::::::..:::.. Target 25 UCUUCAGCACACUGGCAUUU 44
vun-mir2636	TC1711	Beta-1,3-glucanase-like protein	Metabolism	miRNA 19 UAAGUCGU-GUGAUUGUAGG 1 : :::::: ::::::::::: Target 1279 AAUCAGCAACACUAACAUCC 1298
vun-mir2657	TC7897	Proteinase inhibitor 20	Metabolism	miRNA 20 UGUUUUAGUUAUGUUAUUUU 1 :.::::: ::::.::::::: Target 934 AUAAAAUAAAUAUAAUAAAA 953
vun-mir2657	FG852576	Heat shock protein 70 cognate	Stress related	miRNA 22 GUUGUUUUAGUUAUGUUAUUUU 1 :::.:.:::::::. :::.::: Target 77 CAAUAGAAUCAAUGAAAUGAAA 98
vun-mir2657	TC5942	2,4-D inducible glutathione S-transferase	Metabolism	miRNA 21 UUGUUUUAGUUAUGUUAUUUU 1 ::.:::::. ::..::::::: Target 745 AAUAAAAUUUAUGUAAUAAAA 765
vun-mir2678	EF472252	Bound starch synthase	Metabolism	target 5′ U UG UG A 3′ GGC G GCA GAC CUG C CGU UUG miRNA 3′ UG G UG AAAU 5′
vun-mir2678	D88122	CPRD46 protein	Stress related	target 5′ U C G 3′ GCGCGUA CAACUU UGCGCGU GUUGAA miRNA 3′ CUG U AU 5′
vun-mir2678	AY466858	Peroxisomal ascorbate peroxidase	Metabolism	target 5′ U A C A 3′ GGCACG UG CGGC ACUU CUGUGC GC GUUG UGAA miRNA 3′ U AU 5′
vun-mir2678	AB028025	YLD mRNA for regulatory protein	Metabolism	target 5′ A CCA C G 3′ GCGC GCG CGGCGAC UGUG CGC GUUGUUG miRNA 3′ C AAAU 5′
vun-mir2950	TC11773	F-box/Kelch-repeat protein	Transcription factor	miRNA 21 AAGUCAGACGUUCUCUACCUU 1 ::::::::::::::::::::: Target 614 UUCAGUCUGCAAGAGAUGGAA 634
vun-mir2950	TC2831	Ethylene responsive protein	Stress related	miRNA 20 AGUCAGACGUUCUCUACCUU 1 :..:: ::.::::::::::. Target 1700 UUGGUAUGUAAGAGAUGGAG 1719
vun-mir3434	TC7167	Protein transport protein Sec24-like At3g07100	Transporter	miRNA 20 AGAGUACCGACUAUGAGAGU 1 :::.::::.:::: ::.::: Target 662 UCUUAUGGUUGAUUCUUUCA 681
vun-mir4351	TC5899	Expressed protein	Hypothetical protein	miRNA 22 GGUUGAGGUUGACUUGGGAUUG 1 :::::::::::::::::::::: Target 27 CCAACUCCAACUGAACCCUAAC 48
vun-mir4351	FF391835	NADH-ubiquinone oxidoreductase chain 2	Metabolism	miRNA 20 UUGAGGUUGACUUGGGAUUG 1 ::: ::::.: ::::::::. Target 22 AACCCCAAUUAAACCCUAAU 41
vun-mir4392	TC14606	AKIN beta1	Signal transduction	miRNA 22 AGGCUUUAGUGCAAGAGUGUCU 1 : : ::::::::: .:::.::: Target 791 UGCUAAAUCACGUCUUCAUAGA 812
vun-mir4392	TC9038	SNF1-related protein kinase regulatory beta subunit 1	Signal transduction	miRNA 22 AGGCUUUAGUGCAAGAGUGUCU 1 : : ::::::::: .:::.::: Target 979 UGCUAAAUCACGUCUUCAUAGA 1000
vun-mir4408	TC2049	Monooxygenase	Metabolism	miRNA 24 AGGAUAUGAGUAGGUUACAACAAC 1 :: :::.::::: :: ::::::: Target 369 UCAGAUAUUCAUCAAAAGUUGUUG 392
vun-mir4992	FG809835	TfIIE	Transcription factor	miRNA 22 GACUUUUUUUGGUAGAAUCUAC 1 :::::::::::::::::::::: Target 247 CUGAAAAAAACCAUCUUAGAUG 268
vun-mir4992	TC11468	Uncharacterized protein At2g03890.2	Hypothetical protein	miRNA 22 GACUUUUUUUGGUAGAAUCUAC 1 :::::::: :::::.::::::: Target 836 CUGAAAAAUACCAUUUUAGAUG 857
vun-mir4992	TC414	Zinc finger protein 7	Transcription factor	miRNA 22 GACUUUUUUUGGUAGAAUCUAC 1 .:::.:.:::::::.::.::: Target 739 UUGAGAGAAACCAUUUUGGAUC 760
vun-mir4992	TC2268	Zinc finger protein 4	Transcription factor	miRNA 22 GACUUUUUUUGGUAGAAUCUAC 1 .:::.:.:::::::.::.::: Target 857 UUGAGAGAAACCAUUUUGGAUC 878
vun-mir5012	TC1335	Ribosomal protein L30	Structural protein	miRNA 21 CCUUGUGUGCCUCGUCGUUUU 1 ::::.::. :::::::::::: Target 209 GGAAUACGAGGAGCAGCAAAA 229
vun-mir5012	TC59	Acireductone dioxygenase	Metabolism	miRNA 21 CCUUGUGUGCC-UCGUCGUUUU 1 ::::::::: : :::::::::: Target 19 GGAACACACUGUAGCAGCAAAA 40
vun-mir5012	TC12731	Mn-specific cation diffusion facilitator transporter	Transporter	miRNA 20 CUUGUGUGCCUCGUCGUUUU 1 ::.::::::::: :::::. Target 186 GAGCACACGGAGAAGCAAGU 205
vun-mir5043	FF401363	Ran-specific GTPase-activating protein	Transcription factor	miRNA 21 CCACCACGUC-UCUUCCUCUUC 1 : :::::::: :::.::::::: Target 444 GAUGGUGCAGGAGAGGGAGAAG 465
vun-mir5215	FG909052	Ferredoxin I precursor	Metabolism	miRNA 21 UUAGUUGAUCGAGUAGGAGGA 1 ::::::::::::::::::::: Target 179 AAUCAACUAGCUCAUCCUCCU 199
vun-mir5215	GH620837	L-lactate dehydrogenase	Metabolism	miRNA 20 UAGUUGAUCGAGUAGGAGGA 1 :::.:: :::::.::::::: Target 491 AUCGACGAGCUCGUCCUCCU 510
vun-mir5215	TC8326	50S ribosomal protein L21	Structural protein	miRNA 21 UUAGUUGAUCGAGUAGGAGGA 1 :::.::.:.:::.::::::.: Target 943 AAUUAAUUGGCUUAUCCUCUU 963
vun-mir5215	FG849457	Vancomycin resistance protein	Stress related	miRNA 20 UAGUUGAUCGAGUAGGAGGA 1 :::::: .:::::::::.: Target 340 AUCAACAGGCUCAUCCUUCG 359
vun-mir5215	TC6816	General substrate transporter	Transporter	miRNA 21 UUAGUUGAUCGAGUAGGAGGA 1 ::::::::.:::: :.::::: Target 1035 AAUCAACUGGCUC-UUCUCCU 1054
vun-mir5216	FG851044	Metal ion binding	Transcription factor	miRNA 22 AAGGUGACAAAAAGUGAGGGUU 1 : .:::: :::::.:::.:::: Target 227 UAUCACUUUUUUUUACUUCCAA 248
vun-mir5216	FG841236	T5I8.13	Transcription factor	miRNA 22 AAGGUGACAAAAAGUGAGGGUU 1 :::::.: :: ::::.:::::: Target 132 UUCCAUUCUUCUUCAUUCCCAA 153
vun-mir5216	FG931306	Predicted protein	Hypothetical protein	miRNA 21 AGGUGACAAAAAGUGAGGGUU 1 :.:::::::: ::..:.:::: Target 2 UUCACUGUUUCUCGUUUCCAA 22
vun-mir5219	TC16320	Tumor-related protein	Growth and development	miRNA 20 GACGUCGACUCUAAGGUACU 1 ::::: :::::.:: ::::: Target 141 CUGCACCUGAGGUUACAUGA 160
vun-mir5227	TC9947	TINY-like protein	Transcription factor	miRNA 22 AAGAAGUUAGAAGAAGACAAGA 1 ::.::::: ::::::.:::::: Target 1075 UUUUUCAA-CUUCUUUUGUUCU 1095
vun-mir5227	FG842691	HMG1/2-like protein	Transcription factor	miRNA 20 GAAGUUAGAAGAAGACAAGA 1 :::::::.::.:::: ::.: Target 27 CUUCAAUUUUUUUCUAUUUU 46
vun-mir5227	FG886406	Probable intracellular septation protein	Growth & development	miRNA 22 AAGAAGUUAGAAGAAGACAAGA 1 : .::::: :::.::.::::.: Target 48 UGUUUCAACCUUUUUUUGUUUU 69
vun-mir5227	TC17852	Glutathione S-transferase PM24	Metabolism	miRNA 20 GAAGUUAGAAGAAGACAAGA 1 :::::::.:::: :::::: Target 1044 CUUCAAUUUUCUCGUGUUCU 1063
vun-mir5227	TC10272	DNA-directed RNA polymerase subunit	Transcription factor	miRNA 20 GAAGUUAGAAGAAGACAAGA 1 :::::: ::.::.:::::: Target 288 CUUCAAGAUUUUUUUGUUCU 307
vun-mir5241	TC10790	VDAC-like porin	Transporter	miRNA 20 AAGUGAGAAGGUAAGUGGGU 1 ::::::::::::::::..:: Target 201 UUCACUCUUCCAUUCAUUCA 220
vun-mir5241	TC18525	Peptidyl-prolyl cis-trans isomerase	Metabolism	miRNA 20 AAGUGAGAAGGUAAGUGGGU 1 :::..::::::.::::::.: Target 58 UUCGUUCUUCCGUUCACCUA 77
vun-mir5241	FG863193	Probable plastid-lipid-associated protein 13	Stress related	miRNA 20 AAGUGAGAAGGUAAGUGGGU 1 ::::.:: :.:::::::.:: Target 158 UUCAUUCAUUCAUUCACUCA 177
vun-mir5241	TC7362	Serine/threonine protein kinase	Signal transduction	miRNA 20 AAGUGAGAAGGUAAGUGGGU 1 ::..::.:::.:::::..:: Target 934 UUUGCUUUUCUAUUCAUUCA 953
vun-mir5241	TC16629	Multidrug resistance protein	Disease related	miRNA 20 AAGUGAGAAGGUAAGUGGGU 1 :::::::::::: :: :.:: Target 915 UUCACUCUUCCAGUCUCUCA 934
vun-mir5241	TC2781	Non-specific lipid-transfer protein	Transporter	miRNA 20 AAGUGAGAAGGUAAGUGGGU 1 ::::::::::: ::: :.:: Target 20 UUCACUCUUCCUUUCUCUCA 39
vun-mir5241	TC212	Chaperone GrpE type 2	Stress related	miRNA 20 AAGUGAGAAGGUAAGUGGGU 1 ::::.::: .: :::::::: Target 207 UUCAUUCUCUCCUUCACCCA 226
vun-mir5255	TC8912	Pyruvate kinase	Signal transduction	miRNA 20 AGUACAGGAGAUAGGACAGU 1 :.:::::.:::.::.:::.: Target 71 UUAUGUCUUCUGUCUUGUUA 90
vun-mir5255	TC18327	Cysteine protease	Metabolism	miRNA 20 AGUACAGGAGAUAGGACAGU 1 ::: :::::. ::.:::::: Target 605 UCAAGUCCUUGAUUCUGUCA 624
vun-mir5261	FG838847	Chromosome undetermined scaffold_221	Hypothetical protein	miRNA 21 UCGGUUUCGGUAGAUGUUAGC 1 ::::::::::::::::::::: Target 540 AGCCAAAGCCAUCUACAAUCG 560
vun-mir5261	FF398912	TIR	Stress related	miRNA 21 UCGGUUUCGGUAGAUGUUAGC 1 ::::::::.:::::::::::: Target 413 AGCCAAAGUCAUCUACAAUCG 433
vun-mir5290	TC3168	Hydroxyproline-rich glycoprotein	Disease related	miRNA 24 AUACACAGAAAGAGAGAGAUGAAA 1 : : : :::::::::.::::.::: Target 82 UCUCUUUCUUUCUCUUUCUAUUUU 105
vun-mir5290	FG844083	PAS sensor protein	Signal transduction	miRNA 24 AUACACAGAAAGAGAGAGAUGAAA 1 : : : :::::::.:::.::.::: Target 99 UUUCUCUCUUUCUUUCUUUAUUUU 122
vun-mir5290	FG871448	Eco57I restriction endonuclease	Metabolism	miRNA 20 ACAGAAAGAGAGAGAUGAAA 1 : ::::::::::::: :::: Target 42 UCUCUUUCUCUCUCUCCUUU 61
vun-mir5290	TC11392	Ribonuclease III	Transcription factor	miRNA 24 AUACACAGAAAGAGAGAGAUGAAA 1 ::: :: ::: ::::.:.:::::: Target 841 UAUAUGACUUCCUCUUUUUACUUU 864
vun-mir5290	TC12655	Calcium dependent protein kinase	Signal transduction	miRNA 20 ACAGAAAGAGAGAGAUGAAA 1 ::::::.:.:::.:.:::: Target 1254 GGUCUUUUUUUCUUUGCUUU 1273
vun-mir5290	TC4908	ACC oxidase	Growth & development	miRNA 22 ACACAGAAAGAGAGAGAUGAAA 1 : : ::::::::::::::. :: Target 1376 UCUCUCUUUCUCUCUCUAUCUU 1397
vun-mir5290	FG874464	RNA-binding protein	Transcription factor	miRNA 20 ACAGAAAGAGAGAGAUGAAA 1 : ::::::::::::: .::: Target 14 UCUCUUUCUCUCUCUCUUUU 33
vun-mir5298	TC16082	Translation initiation factor IF	Transcription factor	miRNA 25 AAGAAGUAGAAG-UAGAACUUUAGGU 1 : .::::::::: : ::::::::::: Target 34 UCUUUCAUCUUCGAACUUGAAAUCCA 59
vun-mir5298	TC11481	Non-specific lipid-transfer protein	Transporter	miRNA 24 AGAAGUAGAAGUAGAACUUUAGGU 1 :.: ::: ::.:::::::.::..: Target 614 UUUACAUGUUUAUCUUGAGAUUUA 637
vun-mir5298	TC16211	(Iso) Flavonoid glycosyltransferase	Metabolism	miRNA 25 AAGAAGUAGAAGUAGAACUUUAGGU 1 : :: .. :::: :::::::::::: Target 233 UCCUCUGCCUUCUUCUUGAAAUCCA 257
vun-mir5376	TC18575	Zgc:158399 protein	Hypothetical protein	miRNA 23 AGAGUUUAAGAAGUGUUAGAGGU 1 ::::::::::::::::::::::: Target 517 UCUCAAAUUCUUCACAAUCUCCA 539
vun-mir5376	TC16446	Predicted protein	Hypothetical protein	miRNA 23 AGAGUUUAAGAAGUGUUAGAGGU 1 :::::::::::::: :::.: :: Target 687 UCUCAAAUUCUUCAGAAUUUACA 709
vun-mir5376	FC457472	Chromosome chr1 scaffold_135	Hypothetical protein	miRNA 20 GUUUAAGAAGUGUUAGAGGU 1 .: ::::::::::::::.: Target 141 AGAUUUCUUCACAAUCUCUA 160
vun-mir5561	TC1062	H^+^/Ca^2+^ exchanger 2	Transporter	miRNA 20 UGUAAAUCUCUCUCUCUCUA 1 : ::::::::::::::::: Target 8 AGAUUUAGAGAGAGAGAGAG 27
vun-mir5561	TC8162	GTPase	Metabolism	miRNA 20 UGUAAAUCUCUCUCUCUCUA 1 :..: :::::::::::::: Target 102 AUGUAUAGAGAGAGAGAGAG 121
vun-mir5561	TC11798	Cold shock domain	Stress related	miRNA 20 UGUAAAUCUCUCUCUCUCUA 1 ::: : : :::::::::::: Target 2 ACAGUGACAGAGAGAGAGAU 21
vun-mir5758	TC975	Chromosome chr11 scaffold_13	Hypothetical protein	miRNA 21 GUUUAUGUAUCUAGGUUGAAU 1 ::::::::::::::::::::: Target 213 CAAAUACAUAGAUCCAACUUA 233
vun-mir5758	TC5742	Pyrophosphate-dependent phosphofructo-1-kinase	Signal transduction	miRNA 21 GUUUAUGUAUCUAGGUUGAAU 1 .:::::.::::::::::: :: Target 306 UAAAUAUAUAGAUCCAACCUA 326
vun-mir5758	TC16939	Chromosome undetermined scaffold_310	Hypothetical protein	miRNA 20 UUUAUGUAUCUAGGUUGAAU 1 :::::::: :::::::: :: Target 509 AAAUACAUUGAUCCAACGUA 528
vun-mir5770	TC1925	Amine oxidase	Metabolism	miRNA 21 AGUAGGUUUGGUAUCAGGAUU 1 ::::::::::::::::::::: Target 165 UCAUCCAAACCAUAGUCCUAA 185
vun-mir5770	TC5168	Copper amine oxidase	Metabolism	miRNA 21 AGUAGGUUUGGUAUCAGGAUU 1 :..::::::::::::::: :: Target 148 UUGUCCAAACCAUAGUCCAAA 168
vun-mir5770	TC18480	Ribonuclease H	Transcription factor	miRNA 20 GUAGGUUUGGUAUCAGGAUU 1 :::.:::.:.:::::..::: Target 613 CAUUCAAGCUAUAGUUUUAA 632
vun-mir5770	TC1738	Allyl alcohol dehydrogenase	Metabolism	miRNA 20 GUAGGUUUGGUAUCAGGAUU 1 ::::.::::. ::::.::.: Target 766 CAUCUAAACUUUAGUUCUGA 785
vun-mir6252	FG841373	Nucleoporin-like protein	Transcription factor	miRNA 23 UUGGGAGAGAGUUGUGUUGAGUA 1 ::::::::::::::::::::::: Target 24 AACCCUCUCUCAACACAACUCAU 46
vun-mir6252	FG857360	Membrane protein	Transporters	miRNA 21 GGGAGAGAGUUGUGUUGAGUA 1 .::::::::::::: ::::: Target 247 UCCUCUCUCAACACUCCUCAU 267
vun-mir6252	TC15301	Homeobox domain, ZF-HD class	Transcription factor	miRNA 23 UUGGGAGAGAGUUGUGUUGAGUA 1 : : :::::::::: ::::::: Target 9 AUCACUCUCUCAACUCAACUCAA 31
vun-mir7696	FG864277	BZIP transcription	Transcription factor	miRNA 20 AAAACUUAAAUUCAUGAACA 1 :::::::::::::::::::: Target 17 UUUUGAAUUUAAGUACUUGU 36
vun-mir7696	FF383199	Olfactory receptor	Signal transduction	miRNA 20 AAAACUUAAAUUCAUGAACA 1 :::: : :::::::::::: Target 141 UUUUUAUUUUAAGUACUUGG 160
vun-mir8182	TC3507	Pectin methylesterase	Metabolism	miRNA 21 CAGUAGUGUUUGCGUUGUGUU 1 ::::::::::..:::::: :. Target 654 GUCAUCACAAGUGCAACAGAG 674
vun-mir9748	TC16306	Lectin-like protein kinase	Signal transduction	miRNA 22 GAGGAGGGAGUUGUGAAGGAAG 1 : .:::..:::::::::::::. Target 17 CGUCUCUUUCAACACUUCCUUU 38
vun-mir9748	TC1064	Zinc finger, RING-type: Thioredoxin-related	Transcription factor	miRNA 22 GAGGAGGGAGUUGUGAAGGAAG 1 .:::::.:::::: .::.:::: Target 16 UUCCUCUCUCAACUUUUUCUUC 37
vun-mir9748	TC9843	Beta-xylosidase/alpha-L-arabinosidase	Metabolism	miRNA 20 GGAGGGAGUUGUGAAGGAAG 1 :.::..::::::::::::: Target 478 CUUCUUUCAACACUUCCUUG 497
vun-mir9748	TC15743	Heat shock protein	Stress related	miRNA 22 GAGGAGGGAGUUGUGAAGGAAG 1 :::.:::::::::.:: .:::: Target 244 CUCUUCCCUCAACGCUCUCUUC 265
vun-mir9748	TC15591	Transcription factor AHAP2	Transcription factor	miRNA 22 GAGGAGGGAGUUGUGAAGGAAG 1 .::.:::::::: :::::: :: Target 64 UUCUUCCCUCAAGACUUCCAUC 85
vun-mir9748	TC298	Glutathione reductase	Metabolism	miRNA 20 GGAGGGAGUUGUGAAGGAAG 1 .:::.::::::::: .:::: Target 95 UCUCUCUCAACACUCUCUUC 114
vun-mir9748	TC1040	Glycine-rich protein 2b	Transcription factor	miRNA 20 GGAGGGAGUUGUGAAGGAAG 1 ::.:::: .:::::::::: Target 567 ACUUCCUCUGCACUUCCUUC 586

The transcription factor myeloblastosis (MYB) is an important regulator of many developmental and physiological processes in plants. Ballester et al. [53], suggested that the MYB also plays a significant role in regulating the flavonoid pathway in plants. The newly identified cowpea miRNA family vun-834 is found to target the MYB transcription factors. Thus this miRNA is an important resource to fine tune the MYB regulation for the desirable traits in cowpea fruit. The transcription factor, zinc finger is believed to be involved in many biotic and abiotic stresses as responding gene to manage the plant under these stresses [54]. The same family of transcription factor is also reported to play a crucial role in plant development [55]. The newly identified cowpea miRNA families vun-miR834 and 4992 are found to target this zinc finger transcription factor family. These miRNAs are important resources to regulate the zinc finger family proteins for the betterment of cowpea under various biotic and abiotic stresses and fruit development.

Similarly 12% targeted genes by cowpea miRNAs are engaged in transport activities. ATP-binding cassette transporters comprise a highly conserved family of ATP-binding proteins that are involved in transporting of various molecules across plasma membrane. Here vun-miR1514 is identified to target ATP-binding cassette transporters. Such findings are in agreement with the other workers in the miRNA field [37],[43].

Biotic and abiotic stresses like salinity, drought, temperature extremities, heavy metals, pathogen attacks, and pollution cause huge yield reductions in plants [56]. Naturally plants have various systems to protect themselves from these stresses that occur at various levels, i.e., at whole plant, tissue, cellular, sub-cellular, genetic and molecular levels [56]–[60]. Many studies suggest that plant miRNAs are involved in these stresses [9],[17],[61]. In this study identified miRNAs such as vun-miR1525, 2657 and 9748 also targeted heat shock proteins that expressed in response of heat stress. This suggests the role of these miRNAs during the heat stressed condition of plants. Similar findings were reported in switch grass [17].

Some miRNAs of cowpea were observed to target the protein functioning in the process of cell signal transduction. Almost similar findings were observed by many researchers in various organisms [42],[43]. Protein kinases are key regulators of cell function and play crucial role in protein phosphorylation and dephosphorylation that are major signaling pathways induced by osmotic stress in higher plants. Similarly, SNF1 (sucrose non-fermenting-1) is an osmotic-stress-activated protein kinase in *Arabidopsis thaliana* that can significantly impact drought tolerance of *Arabidopsis thaliana* plants [62]. These two important proteins were targeted by cowpea miRNAs families, like vun-miR435, 2606, 2609 and 4392 respectively. Serine/threonine protein kinase **(**STPKs) is another protein kinase that is targeted by miRNA family (miR5241), act as sensors of environmental signals and regulate different developmental changes and also host pathogen interactions [63].

In this study, newly profiled cowpea miRNAs were also observed to target hypothetical proteins, growth and development, structural proteins and disease related proteins. Such findings were also published earlier [19],[21],[37].

## 4. Conclusion

The current study is resulted 46 new miRNAs and their 138 targeted genes in an important commercial plant cowpea. All these miRNAs are profiled for the first time in cowpea. These findings will serve as resources to fine tune cowpea plant at micro-molecular level. This will help us to enhance the production ability of cowpea against biotic and abiotic stress tolerance. Furthermore these miRNAs and their targets are also powerful functional genomic resources in the Kingdom plantae.
